# Caloric Restriction and Hypothalamic Leptin Gene Therapy Have Differential Effects on Energy Partitioning in Adult Female Rats

**DOI:** 10.3390/ijms22136789

**Published:** 2021-06-24

**Authors:** Russell T. Turner, Carmen P. Wong, Kristina M. Fosse, Adam J. Branscum, Urszula T. Iwaniec

**Affiliations:** 1 Skeletal Biology Laboratory, School of Biological and Population Health Sciences, Oregon State University, Corvallis, OR 97331, USA; russell.turner@oregonstate.edu (R.T.T.); carmen.wong@oregonstate.edu (C.P.W.); fossek@oregonstate.edu (K.M.F.); 2 Biostatistics Program, School of Biological and Population Health Sciences, Oregon State University, Corvallis, OR 97331, USA; adam.branscum@oregonstate.edu

**Keywords:** obesity, marrow adipose tissue, white adipose tissue, *Ucp-1*, leptin, adiponectin, bone architecture

## Abstract

Dieting is a common but often ineffective long-term strategy for preventing weight gain. Similar to humans, adult rats exhibit progressive weight gain. The adipokine leptin regulates appetite and energy expenditure but hyperleptinemia is associated with leptin resistance. Here, we compared the effects of increasing leptin levels in the hypothalamus using gene therapy with conventional caloric restriction on weight gain, food consumption, serum leptin and adiponectin levels, white adipose tissue, marrow adipose tissue, and bone in nine-month-old female Sprague-Dawley rats. Rats (*n* = 16) were implanted with a cannula in the 3rd ventricle of the hypothalamus and injected with a recombinant adeno-associated virus, encoding the rat gene for leptin (rAAV-*Lep*), and maintained on standard rat chow for 18 weeks. A second group (*n* = 15) was calorically-restricted to match the weight of the rAAV-*Lep* group. Both approaches prevented weight gain, and no differences in bone were detected. However, calorically-restricted rats consumed 15% less food and had lower brown adipose tissue *Ucp-1* mRNA expression than rAAV-*Lep* rats. Additionally, calorically-restricted rats had higher abdominal white adipose tissue mass, higher serum leptin and adiponectin levels, and higher marrow adiposity. Caloric restriction and hypothalamic leptin gene therapy, while equally effective in preventing weight gain, differ in their effects on energy intake, energy expenditure, adipokine levels, and body composition.

## 1. Introduction

Normal weight gain during middle age (0.25–0.50 kg/year) is associated with increased risk for several chronic diseases, most notably type II diabetes [1,2]. Hypertension, cardiovascular disease and certain cancers are also positively associated with weight gain [3]. The underlying mechanisms leading to the energy imbalance responsible for normal weight gain in adults are not well understood, but incremental development of leptin resistance, associated with higher circulating leptin, is thought to be a contributing factor [4]. In support, decreases in serum leptin levels in overweight individuals result in improved leptin sensitivity [5]. Whatever the precise mechanism(s) of action, caloric restriction, sufficient to result in a negative energy balance, is a common strategy to lose excess weight and/or mitigate health risks associated with weight gain.

The increase in population level body mass index noted in recent decades is also paralleled by increased prevalence of dieting as a form of voluntary caloric restriction. Prevalence of dieting varies with age, sex, economic status, and ethnicity, but in a recent study, 57–63% of middle-aged women (35–65 years old) dieted to control weight [6,7,8,9]. Weight loss due to caloric restriction may attenuate or reverse some of the health risks associated with excessive weight gain, especially in younger individuals. However, the long-term efficacy of conventional weight loss interventions is generally poor, and many individuals weight-cycle through repetitive bouts of weight loss followed by rapid weight regain [10,11,12]. In addition to likely beneficial effects, caloric restriction has the potential for detrimental side effects. For example, weight loss in adults is often associated with bone loss and, as a consequence, increased risk for osteoporosis [13]. In adult male rats, weight loss induced by caloric restriction resulted in bone loss due to a combination of increased bone resorption and decreased bone formation [14]. In some populations, intentional weight loss is associated with increased mortality [15,16,17]. Thus, preventing weight gain by maintaining a neutral energy balance during adulthood should be a primary strategy for long duration weight control.

Although a pleotropic hormone with peripheral and central activity, leptin’s actions as a regulator of energy balance are primarily centrally mediated [18,19]. Importantly, increasing hypothalamic leptin levels bypasses leptin resistance at the blood brain barrier and, compared to *ad lib*-fed or pair-fed controls, reduces weight gain, and lowers peripheral leptin levels in rodents consuming normal and high fat diets [20,21,22,23]. We previously evaluated the long-duration effects of increasing hypothalamic leptin levels using gene therapy (rats injected in 3rd ventricle of their hypothalamus with recombinant adeno-associated virus (rAAV) encoding the rat gene for leptin) on energy balance and bone metabolism in 9-month-old female rats [24]. At study termination, rAAV-*Lep* treated rats weighed less, had less abdominal white adipose tissue (WAT), and had lower serum leptin compared to rats administered the control vector rAAV-GFP (green florescent protein). These findings suggest that interventions targeted toward increasing leptin sensitivity have the potential to overcome leptin resistance and, thereby, prevent undesirable weight gain. Leptin resistance can be reduced by lowering serum leptin levels [5] or by directly increasing hypothalamic leptin levels using gene therapy [20]. However, it is not clear whether these two approaches illicit similar adaptive responses.

The primary objective of the present study was to compare (1) the effects of increasing hypothalamic leptin gene expression with gene therapy to (2) conventional caloric restriction on weight gain, food consumption, serum leptin and adiponectin levels, abdominal adiposity, marrow adiposity, *Ucp-1* gene expression (as an index of non-shivering thermogenesis) in brown adipose tissue (BAT) and bone. The 18-week study was performed in 9-month-old female rats fed a normal diet to model prevention of age-related weight gain common in healthy adults. The results show that hypothalamic leptin gene therapy and caloric restriction, while both effective in preventing weight gain, differ in their effects on energy partitioning.

## 2. Results

### 2.1. Effects of Hypothalamic rAAV-Lep or Caloric Restriction on Body Weight and Food Consumption

**Figure 1:**
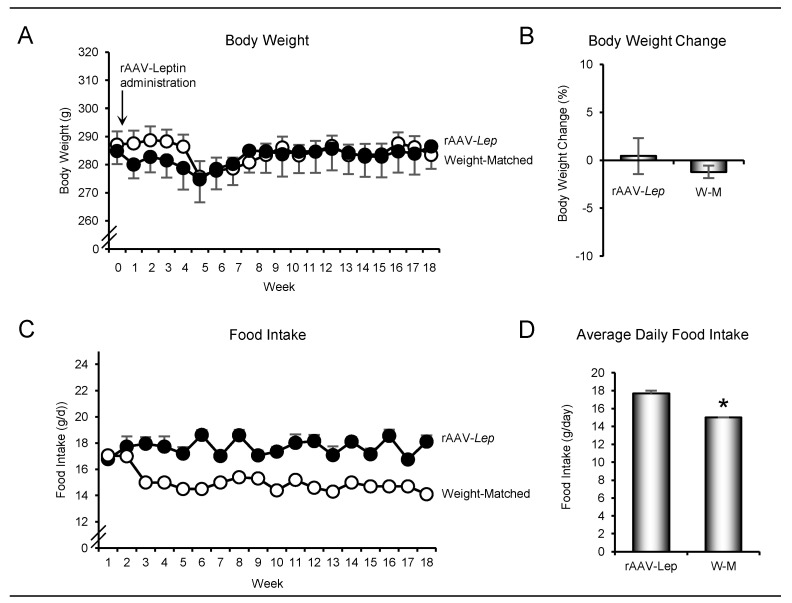
Effects of hypothalamic leptin gene therapy (rAAV-*Lep*) and caloric restriction (mean 15% restriction to weight-match rAAV-*Lep* treated animals; weight-matched, W-M) on (**A**) body weight as a function of time, (**B**) body weight change between initiation and termination of treatment, (**C**) food intake/day as a function of time, and (**D**) average daily food intake. Data are mean ± SE; *n* = 15–16/group. * Different from rAAV-*Lep*, *p* ≤ 0.05.

The effects of hypothalamic rAAV-*Lep* gene therapy and weight matching using caloric restriction on body weight as a function of time, net body weight change during the 18 weeks of treatment, food intake/day as a function of time, and average daily food intake are shown in Figure 1. As intended, body weight did not differ with time between the rAAV-*Lep* and calorically-restricted weight-matched rats (Figure 1A). Weight in the rAAV-*Lep* rats did not differ from baseline weight at any time point during the 18-week duration of study. Final body weight did not change significantly compared to baseline values (286 ± 3 g) for either group of rats (Figure 1B). These findings indicate that rAAV-*Lep* stabilized body weight in adult rats without reducing food intake. In contrast, the weight-matched rats consumed less food (Figure 1C); average daily food consumption was 15% lower in weight-matched rats compared to rAAV-*Lep* rats (Figure 1D).

### 2.2. Effects of Hypothalamic rAAV-Lep or Caloric Restriction on Abdominal WAT, Serum Adipokines and Tissue Gene Expression

**Figure 2:**
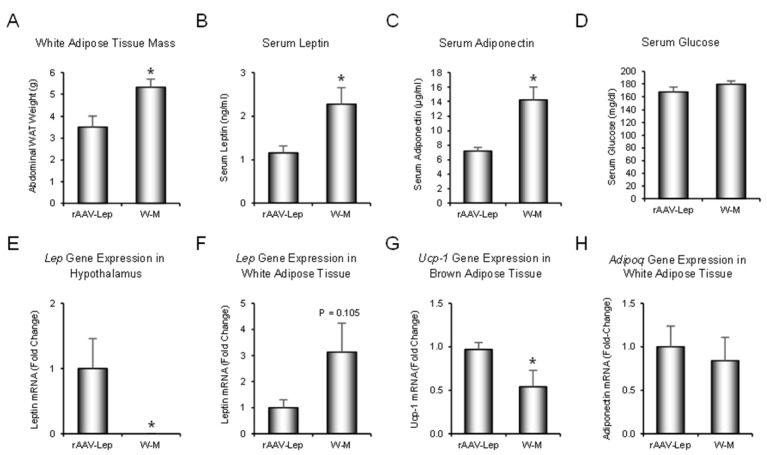
Effects of hypothalamic leptin gene therapy (rAAV-*Lep*) and caloric restriction (mean 15% restriction to weight-match rAAV-*Lep* treated animals; weight-matched, W-M) on (**A**) abdominal white adipose tissue (WAT) mass, (**B**) serum leptin, (**C**) serum adiponectin, (**D**) serum glucose, (**E**) *Lep* gene expression in hypothalamus, (**F**) *Lep* gene expression in abdominal WAT, (**G**) *Ucp-1* gene expression in brown adipose tissue (BAT), and (**H**) *Adipoq* gene expression in WAT. Data are mean ± SE; *n* = 15–16/group for WAT, 14–15/group for leptin, 11–12/group for adiponectin and glucose, 5–12/group for hypothalamic *Lep* expression, 5–7/group for WAT *Lep* expression, 5–11/group for BAT *Ucp-1* expression, and 7/group for WAT *Adipoq* expression. * Different from rAAV-*Lep*, *p* ≤ 0.05.

The effects of rAAV-*Lep* treatment and weight-matching on abdominal WAT mass, serum leptin levels, serum adiponectin levels, serum glucose levels, and steady-state mRNA levels for leptin (*Lep*) in hypothalamus and in abdominal WAT, *Ucp-1* (*Ucp-1*) in BAT, and adiponectin (*Adipoq*) in WAT are shown in Figure 2. Calorically-restricted weight-matched rats had higher WAT mass (Figure 2A), higher serum leptin (Figure 2B), and higher serum adiponectin (Figure 2C). Serum glucose levels did not differ between treatment groups (Figure 2D). As expected, mRNA levels for leptin in hypothalamus were lower (below limit of reliable detection) in weight-matched rats compared to rAAV-*Lep* rats (Figure 2E). mRNA levels for leptin in abdominal WAT tended (*p* = 0.105) to be higher in weight-matched rats (Figure 2F). mRNA levels for *Ucp-1* in BAT were lower in weight-matched rats (Figure 2G). Significant differences between the two groups were not detected in mRNA levels for adiponectin in WAT (Figure 2H). These findings indicate that, although weight matching using caloric restriction and rAAV-*Lep* treatment are equally effective in stabilizing body weight in adult female rats, the metabolic response to the two treatments differs. Specifically, weight matched animals required less food to achieve weight stability than rAAV-*Lep* treated rats.

### 2.3. Effects of Hypothalamic rAAV-Lep or Caloric Restriction on Bone Marrow Adiposity

**Figure 3:**
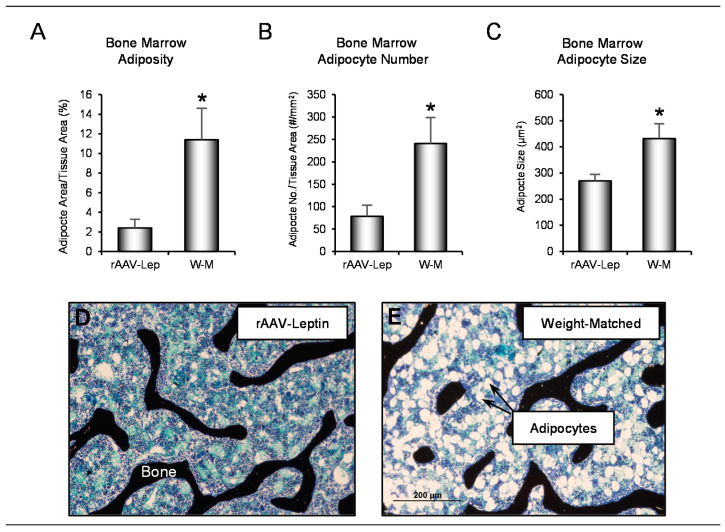
Effects of hypothalamic leptin gene therapy (rAAV-*Lep*) and caloric restriction (mean 15% restriction to weight-match rAAV-*Lep* treated animals; weight-matched, W-M) on (**A**) bone marrow adiposity, (**B**) adipocyte number, and (**C**) adipocyte size in proximal tibia. Representative images from an rAAV-*Lep* treated and a weight matched rat are shown in (**D**,**E**), respectively. Data are mean ± SE; *n* = 5–8/group. * Different from rAAV-*Lep*, *p* ≤ 0.05.

The effects of rAAV-*Lep* treatment and weight-matching on bone marrow adipose tissue (bMAT), in proximal tibia metaphysis, are shown in Figure 3. Adipocyte area/tissue area (Figure 3A), adipocyte number (Figure 3B), and adipocyte size (Figure 3C) were higher in weight-matched rats. Representative images depicting differences in marrow adiposity in the two treatment groups are shown in Figure 3D,E. These findings indicate that, compared to weight matching using caloric restriction, rAAV-*Lep* treatment results in lower bone marrow adiposity.

### 2.4. Effects of Hypothalamic rAAV-Lep or Caloric Restriction on Bone Mass, Architecture, and Serum Markers of Bone Turnover

The effects of rAAV-*Lep* treatment and weight-matching on total femur bone area, bone mineral content, and bone mineral density, measured by dual energy x-ray absorptiometry (DXA), and on bone microarchitecture in femur diaphysis, metaphysis and epiphysis measured by microcomputed tomography (µCT) are shown in Table 1. Significant differences between rAAV-*Lep* and calorically-restricted weight-matched rats were not detected for any of the endpoints measured.

**Figure 4:**
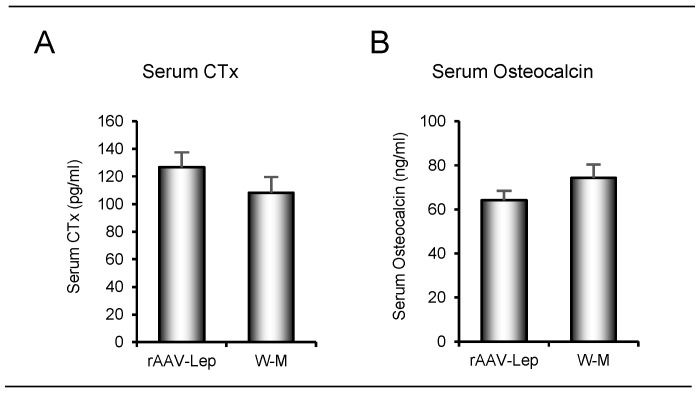
Effects of hypothalamic leptin gene therapy (rAAV-*Lep*) and caloric restriction (mean 15% restriction to weight-match rAAV-*Lep* treated animals; weight-matched, W-M) on (**A**) serum CTX, a marker of global bone resorption and (**B**) serum osteocalcin, a marker of global bone formation. Data are mean ± SE; *n* = 9–11/group.

The effects of rAAV-*Lep* treatment and weight-matching on serum CTX, a marker of global bone resorption, and serum osteocalcin, a marker of global bone formation, are shown in Figure 4. Significant differences between rAAV-*Lep* and calorically-restricted weight-matched rats were not detected for either of the endpoints measured.

## 3. Discussion

The effects of 18 weeks of hypothalamic rAAV-*Lep* gene therapy and involuntary caloric restriction to equalize body weight on food intake, abdominal adipose tissue, serum leptin and adiponectin, bone marrow adipose tissue, and bone were evaluated in 9-month-old female Sprague-Dawley rats. rAAV-*Lep* treated rats maintained body weight at pretreatment levels and calorically-restricted rats did not differ in weight from rAAV-*Lep* treated rats, indicating success of weight matching. Compared to rAAV-*Lep* treated rats, weight matched rats consumed 15% less food, had lower BAT *Ucp-1* mRNA expression and, by experimental design, lower hypothalamic *Lep* mRNA expression. Calorically-restricted weight matched rats also differed from rAAV-*Lep* treated animals in having higher WAT mass, higher bone marrow adiposity, and higher serum leptin and adiponectin levels. Femur bone mineral density, bone microarchitecture, and global markers of bone turnover in serum did not differ between rAAV-*Lep* and calorically-restricted rats.

The effects of obesogenic diets (diets where fat typically contributes 45–60% of energy; this contrasts with normal rodent chow, which contains ~10% of energy from fat) on weight gain and health outcomes have been extensively studied in rodents. These diets are typically used to induce rapid weight gain. However, in wealthy countries, energy intake in excess of the range required to achieve energy balance, rather than a preponderance of dietary fat, is the major driver of weight gain in most adults. Bone elongation ceases in female Sprague-Dawley rats at ~8 months of age [25], indicating cessation of linear growth. However, as in humans, body weight continues to increase during adulthood, even when rats are fed a normal diet [26]. In a prior study, female Sprague-Dawley rats fed a normal diet increased in weight by 16% between 8 and 12 months of age [27]. It is well established that weight loss induced by severe voluntary caloric restriction results in adaptive metabolic responses that act to diminish the magnitude of the resulting negative energy balance. These adaptive responses are believed to contribute to post-diet intervention weight regain [28]. However, it is less clear whether milder caloric restriction, used to promote weight stabilization in adults by achieving a neutral energy balance, results in similar adaptive responses.

In the current study, mild caloric restriction and hypothalamic rAAV-*Lep* treatment were similarly effective in preventing weight gain, but there were metabolic differences in how the treatments led to weight stabilization. Increasing leptin levels in leptin-deficient humans and mice decreases weight, primarily, by reducing appetite, but there is evidence, at least in rodents, that leptin also increases energy expenditure [29]. In this regard, hypothalamic leptin gene therapy in rats able to generate leptin in WAT was shown to stabilize body weight without decreasing appetite [24]. Thus, rAAV-*Lep* treatment prevents weight gain, at least in part, by mechanisms not requiring a decrease in appetite. Weight maintenance using caloric restriction results in lower energy intake compared to hypothalamic rAAV-*Lep* gene therapy, which is a limitation for effective long duration weight control, at least in animal models, because food consumption increases following a return to *ad lib* feeding [30].

The higher *Ucp-1* gene expression levels in BAT suggest that rAAV-*Lep* treatment increases energy expenditure, at least in part, by increasing non-shivering thermogenesis, a finding in agreement with numerous studies [22,31,32]. Interestingly, rAAV-*Lep* treatment not only decreased abdominal WAT mass compared to mild caloric restriction, but it may have decreased steady state mRNA levels for leptin in WAT. Thus, in addition to decreasing fat mass, increasing leptin in the hypothalamus may reduce serum leptin by decreasing leptin production by adipocytes. However, this possibility requires verification.

The peptide hormones adiponectin and leptin are primarily, if not exclusively, synthesized by adipocytes, but their pattern of secretion often differs. Excess weight gain is associated with elevated circulating leptin and decreased circulating adiponectin [33]. Physiologically, this may be important because adiponectin, as well as leptin, can act to increase insulin sensitivity and/or function as immune system modulators [34]. We were, therefore, initially surprised to find that rAAV-*Lep* treatment, while decreasing abdominal WAT mass and serum leptin compared to caloric restriction, also resulted in lower levels of serum adiponectin. Individuals with anorexia have elevated serum adiponectin in spite of very low WAT. It has been suggested that adiponectin produced by bMAT, which typically increases during severe caloric restriction, contributes to high adiponectin levels [35]. If this interpretation is correct, the higher bMAT may be responsible, at least in part, for the higher serum adiponectin observed, in the current study, in calorically-restricted rats.

Decreased energy expenditure following weight loss, even after accounting for the change in body mass, is observed in humans and may contribute to the poor success of dieting as a strategy for long-duration weight maintenance [36]. As shown here, and in previous studies, increasing hypothalamic leptin levels prevents weight gain by mechanisms that do not require reduced appetite. In contrast, prevention of weight gain, in the weight matched rats, required 15% reduction in food, totaling 286 g over the 18-week study. This large difference in energy intake necessary for weight stability, combined with the observed lower WAT mass and higher *Ucp1* mRNA levels in BAT, strongly suggest that rAAV-*Lep* prevents weight gain, in part, by increasing energy expenditure. These findings provide support for the possibility that increasing hypothalamic leptin levels in human subjects, following massive weight loss, can act to facilitate weight maintenance [37,38,39].

Leptin signaling is required for normal skeletal maturation; leptin deficiency in growing mice results in reduced longitudinal bone growth, osteopenia, and osteopetrosis [40,41,42,43,44,45]. Skeletal abnormalities are also observed in leptin receptor-deficient *db/db* mice and *fa/fa* rats [46,47], and these abnormalities are likely responsible for the decreased bone strength observed in leptin-deficient and leptin receptor-deficient rodents [48,49]. Severe caloric restriction, resulting in rapid weight loss, results in bone loss in rats [14]. However, diet-mediated modulation of serum leptin levels associated with moderate weight gain had no effect on bone mass, microarchitecture, or turnover in skeletally mature rats [26]. Similarly, in the present study, increasing hypothalamic leptin levels via rAAV-*Lep* gene therapy prevented the anticipated age-related weight gain but had no effect on femur mass, density, or microarchitecture compared to caloric restriction. The present study, performed in normal rats, and prior studies, performed in *ob/ob* mice, suggest that, although extremely important, the actions of leptin to facilitate normal bone growth and turnover occur at low circulating levels of the hormone, which were achieved in the present study [19,50].

This study was performed in ovary-intact female rats. This is a limitation because gonadal hormone insufficiency, induced by ovariectomy in rats or following menopause in women, accelerates normal age-related total weight gain and/or adipose tissue gain [51,52]. Additionally, age-associated weight gain is common in males of both species. These limitations are somewhat addressed by studies demonstrating the effectiveness rAAV-*Lep* in reducing weight gain in adult male and adult ovariectomized rodents fed normal or high fat diets [22,53,54]. An additional limitation is delivering a viral vector into the hypothalamus to control normal weight gain. Therefore, this study should be viewed as a proof of principle. Future research is encouraged to identify less invasive alternative approaches to increase the transport of leptin across the blood brain barrier to further test the hypothesis that increasing the concentration of leptin in the hypothalamus can overcome leptin resistance.

In summary, caloric restriction and increasing hypothalamic leptin, via rAAV-*Lep* gene therapy, were equally effective in weight maintenance but elicited different metabolic adaptations. The former led to greater adipose tissue storage in abdominal WAT and bone marrow, and the latter stabilized weight without reducing appetite. Future research should focus on elucidating the precise mechanism responsible for these effects. Reduction in metabolic rate, associated with diet-induced weight loss, is considered to be a major cause for post-diet weight regain. Our findings suggest that elevating hypothalamic leptin levels would be a more effective strategy than dieting for long term weight maintenance.

## 4. Materials and Methods

### 4.1. Experimental Design

Nine-month-old female Sprague-Dawley rats were obtained from Harlan (Indianapolis, IN, USA) and maintained under specific pathogen-free conditions in accordance with the NIH Guide for the Care and Use of Laboratory Animals. Nine-month-old rats were used because linear growth has ceased [25], but the animals continue to increase slowly in weight [55], mimicking age-related weight gain in humans. The rats were housed individually in a temperature (21–23 °C) and light-controlled room; lights were on from 6 a.m. to 6 p.m. The experimental protocol was approved (IACUC #D642; 1-13-2004) by the Institutional Animal Care and Use Committee at the University of Florida.

The rats were randomized, by weight, into one of two treatment groups: rAAV-*Lep* (*n* = 16) and weight-matched (*n* = 15). The rAAV-*Lep* rats were implanted with a cannula in the 3rd ventricle of the hypothalamus and injected with rAAV-*Lep* as detailed below. The weight-matched group was calorically-restricted to match the weight of the rAAV-*Lep* group. The rats were weighed and food consumption (standard rat chow) was determined weekly for the 18-week duration of study. 18 weeks represents ~15% of the expected adult lifespan in this animal model.

### 4.2. rAAV Vector Administration

The rAAV-*Lep* vector was constructed, packaged, and administered as described [22,31,32,56,57,58]. To summarize, the vector pTR-CBA-Ob EcoRI fragment of pCR-rOb containing rat leptin cDNA was subcloned into rAAV vector plasmid pAAVβGEnh after deleting the EcoRI fragment carrying the β-glucoronidase cDNA sequence. The rats were anesthetized and stereotaxically implanted with a permanent cannula in the 3rd cerebroventricle of the hypothalamus. The coordinates employed for microinjector placement were determined using the rat brain atlas. After 1 week of recovery, rats were injected intracerebroventricularly with 7.7 × 10^11^ virus particles in 5 µL. Localization and biological response to rAAV-*Lep* vector administration into the 3rd cerebroventricle of the hypothalamus of rats are documented [20,31,32].

### 4.3. Tissue Collection at Necropsy

Overnight fasted (12–16 h) rats were anesthetized with 2–3% isoflurane and blood collected from the abdominal aorta. The animals were then euthanized by decapitation. Serum was frozen at −20 °C for analysis of leptin, adiponectin, glucose, CTX, and osteocalcin. Brains were removed and hypothalamus excised and stored in RNAlater (Ambion, Austin, TX, USA) for analysis of *Lep* gene expression. Abdominal WAT (mesenteric and retroperitoneal) was excised and weighed. Samples of WAT were stored in RNAlater for analyses of *Lep* and *Adipoq* gene expression. Interscapular BAT was removed and stored in RNAlater for analysis of *Ucp-1* gene expression. Femora and tibiae were collected and stored in 70% ethanol for analysis of bone mass, density, microarchitecture (femur) and bone marrow adiposity (tibia).

### 4.4. RNA Analysis

Total cellular RNA was isolated from the entire hypothalamus, abdominal WAT, and interscapular BAT, as described [24]. cDNA for RT-PCR was synthesized using SuperScript First-Strand Synthesis System for RT-PCR (Invitrogen, Carlsbad, CA, USA) and analyzed using primers for rat 18S ribosomal (18S), leptin and *Ucp-1* RNA as described [24]. A standard curve, generated from serial dilutions of purified plasmid DNA that encoded the respective genes, was used to measure mRNA transcript copy number. mRNA data represent normalized copy number using the 18S ribosomal RNA gene. Predesigned KiCqStart SYBR Green rat adiponectin primers were purchased from Sigma (St Louis, MO, USA).

### 4.5. Serum Assays

Serum leptin, adiponectin, glucose, CTX, and osteocalcin were measured as described [24,59].

### 4.6. Histomorphometry

For evaluation of bone marrow adiposity in proximal tibia, longitudinal sections (5 µm thick) were cut on a Jung Reichart microtome as described [60]. Measurements were performed in unstained sections under ultraviolet illumination in a sampling site located in the metaphysis, immediately below the growth plate. All measurements were performed with the OsteoMeasure Image Analysis system (OsteoMetrics, Inc., Decatur, GA, USA). Bone marrow adiposity (adipocyte area/tissue area, %), adipocyte number (number/tissue area, #/mm^2^), and adipocyte size (µm^2^) were determined as described [61].

### 4.7. Densitometry

Total femur bone mineral content (BMC, g), bone area (cm^2^), and bone mineral density (BMD, g/cm^2^) were measured using DXA (Piximus 2, Lunar Corporation, Madison, WI, USA).

### 4.8. Microcomputed Tomography

µCT was used for nondestructive three-dimensional evaluation of bone architecture. Femurs were scanned using a Scanco µCT40 scanner (Scanco Medical AG, Basserdorf, Switzerland) at a voxel size of 16 × 16 × 16 µm and evaluated at a threshold of 245 (gray scale, 0–1000). Cortical bone was evaluated at the midshaft, and cancellous bone was evaluated in the distal femoral metaphysis and epiphysis. For the femoral midshaft, 20 slices (0.32 mm) of bone were evaluated, and (1) total cross-sectional tissue volume (cortical and marrow volume, mm^3^), (2) cortical volume (mm^3^), (3) marrow volume (mm^3^), and (4) cortical thickness (μm) were measured. Polar moment of inertia (mm^4^) was determined as a surrogate measure of bone strength in torsion. For the distal femoral metaphysis, 75 slices (1.2 mm) of bone 150 slices (2.4 mm) proximal to the growth plate were measured and included secondary spongiosa only. For the distal femur epiphysis 60 ± 1 slices (0.96 ± 0.01 mm; entire cancellous compartment) were evaluated. Direct cancellous bone measurements included: (1) cancellous bone volume/tissue volume (%), (2) connectivity density (mm^−3^), (3) trabecular thickness (μm), (4) trabecular number (mm^−1^), and (4) trabecular separation (μm).

### 4.9. Statistical Analysis

Data from adult female rats, randomized to either the rAAV-*Lep* group or a weight-matched calorically-restricted control group, were analyzed to compare mean outcomes using t-tests (with or without equal variance) or the Wilcoxon-Mann-Whitney test. The decision to use a parametric or nonparametric test was based on quantile-quantile plots and the Anderson-Darling test to assess normality. Residual analysis and Levene’s test were used to assess homogeneity of variance. The time course data in Figure 1 was analyzed using repeated measures ANOVA. The Benjamini and Hochberg method, for maintaining the false discovery rate at 5%, was used to adjust for multiple comparisons [62]. Differences were considered significant at *p* ≤ 0.05. All data are presented as mean ± SE. Data analysis was performed using R version 3.6.3

## Figures and Tables

**Table 1:** Effects of hypothalamic leptin gene therapy (rAAV-*Lep*) and caloric restriction (mean 15% restriction to weight-match rAAV-*Lep* treated animals) on bone area, bone mineral content and bone mineral density in total femur and on cortical bone architecture in the femur diaphysis and cancellous bone architecture in the distal femur metaphysis and epiphysis.

	rAAV-*Lep*			Weight-Matched			*p* Value
							
**Dual Energy X-ray Absorptiometry**							
							
**Total Femur**							
Bone area (cm^2^)	2.32 ± 0.03			2.36 ± 0.03			0.592
Bone mineral content (g)	0.462 ± 0.009			0.469 ± 0.008			0.762
Bone mineral density (g/cm^2^)	0.199 ± 0.002			0.198 ± 0.002			0.918
							
**Microcomputed Tomography**							
							
**Midshaft Femur** (cortical bone)							
Cross-sectional volume (mm^3^)	3.27 ± 0.07			3.25 ± 0.06			0.853
Cortical volume (mm³)	2.18 ± 0.04			2.12 ± 0.03			0.464
Marrow volume (mm³)	1.09 ± 0.04			1.13 ± 0.04			0.762
Cortical thickness (µm)	765 ± 9			741 ± 9			0.067
Polar moment of inertia (mm^4^)	16.67 ± 0.67			16.18 ± 0.54			0.762
							
**Distal Femur Metaphysis** (cancellous bone)							
Bone volume/tissue volume (%)	20.0 ± 1.3			23.1 ± 1.2			0.258
Connectivity density (mm^−3^)	63.6 ± 4.4			67.1 ± 4.6			0.762
Trabecular thickness (µm)	70 ± 2			75 ± 2			0.513
Trabecular number (1/mm)	3.9 ± 0.1			4.0 ± 0.1			0.277
Trabecular spacing (µm)	253 ± 7			243 ± 7			0.547
							
**Distal Femur Epiphysis** (cancellous bone)							
Bone volume/tissue volume (%)	38.5 ± 0.7			38.0 ± 0.7			0.762
Connectivity density (mm^−3^)	34.9 ± 1.3			33.6 ± 1.0			0.718
Trabecular thickness (µm)	103 ± 2			102 ± 1			0.814
Trabecular number (mm^−1^)	3.7 ± 0.1			3.7 ± 0.1			0.762
Trabecular spacing (µm)	256 ± 6			260 ± 5			0.762

Data are mean ± SE; *n* = 14–16/group.

## Data Availability

All relevant data are included within the manuscript. Raw data will be made available from the corresponding author upon reasonable request.

## Abbreviations

BAT	Brown adipose tissue
bMAT	Bone marrow adipose tissue
DXA	Dual energy x-ray absorptiometry
GFP	Green fluorescent protein
µCT	Microcomputed tomography
rAAV	Recombinant adeno-associated virus
*Ucp-1*	Uncoupling protein 1
W-M	Weight-matched
WAT	White adipose tissue
